# Association of sleep complaints with all-cause and heart disease mortality among US adults

**DOI:** 10.3389/fpubh.2023.1043347

**Published:** 2023-03-21

**Authors:** Qiao Wang, Shimin Hu, Na Clara Pan, Tingting Zhang, Liankun Ren, Yuping Wang

**Affiliations:** ^1^Department of Neurology, Xuanwu Hospital, Capital Medical University, Beijing, China; ^2^National Center for Neurological Disorders, Beijing, China; ^3^Beijing Key Laboratory of Neuromodulation, Beijing, China; ^4^Institute of Sleep and Consciousness Disorders, Center of Epilepsy, Beijing Institute for Brain Disorders, Capital Medical University, Beijing, China

**Keywords:** sleep complaint, sleep duration, sleep disorder, all-cause mortality, heart disease mortality

## Abstract

**Introduction:**

Compared with sleep disorders, no consensus has been reached on whether a subjective complaint of having trouble sleeping is associated with increased all-cause and heart disease mortality risk. Previous studies displayed considerable heterogeneity in population disease characteristics and duration of follow-up. Therefore, the aims of this study were to examine the relationship between sleep complaints and all-cause and heart disease mortality and whether the associations were influenced by follow-up time and population disease characteristics. In addition, we aimed to figure out the influence of the joint effects of sleep duration and sleep complaints on mortality risk.

**Methods:**

The present study utilized data from five cycles of the National Health and Nutrition Examination Survey (NHANES) (2005~2014) linked with the most updated 2019 National Death Index (NDI). Sleep complaints were determined by answers to “Have you ever told a doctor or other health professional that you have trouble sleeping?” and “Have you ever been told by a doctor or other health professional that you have a sleep disorder?”. Those who answered ‘Yes' to either of the aforementioned two questions were considered as having sleep complaints.

**Results:**

A total of 27,952 adult participants were included. During a median follow-up of 9.25 years (interquartile range, 6.75–11.75 years), 3,948 deaths occurred and 984 were attributable to heart disease. A multivariable-adjusted Cox model revealed that sleep complaints were significantly associated with all-cause mortality risk (HR, 1.17; 95% CI, 1.07–1.28). Subgroup analysis revealed that sleep complaints were associated with all-cause (HR, 1.17; 95% CI, 1.05–1.32) and heart disease (HR, 1.24; 95% CI, 1.01–1.53) mortality among the subgroup with cardiovascular disease (CVD) or cancer. In addition, sleep complaints were more strongly associated with short-term mortality than long-term mortality. The joint analysis of sleep duration and sleep complaints showed that sleep complaints mainly increased the mortality risk in those with short (< 6 h/day, sleep complaints HR, 1.40; 95% CI, 1.15–1.69) or recommended (6–8 h/day, sleep complaints HR, 1.15; 95% CI, 1.01–1.31) sleep duration group.

**Discussion:**

In conclusion, sleep complaints were associated with increased mortality risk, indicating a potential public benefit of monitoring and managing sleep complaints in addition to sleep disorders. Of note, persons with a history of CVD or cancer may represent a potentially high-risk group that should be targeted with a more aggressive intervention of sleep problems to prevent premature all-cause and heart disease death.

## 1. Introduction

Sleep is essential for the maintenance of human life (1). Sleep disorders typically cause disturbances in the quality and duration of sleep, resulting in impaired daytime functioning and distress. Sleep disorders can be divided into seven major categories according to the International Classification of Sleep Disorders, third edition (ICSD-3) (2), which include insomnia disorders, sleep-related breathing disorders, central disorders of hypersomnolence, circadian rhythm sleep–wake disorders, sleep-related movement disorders, parasomnias, and other sleep disorders. Insomnia and obstructive sleep apnea are the two most common sleep disorders, with approximately 6–23% (3, 4) and 6–17% (5) of the general population fulfilling the diagnostic criteria of insomnia and moderate to severe obstructive sleep apnea, respectively. Sleep disorders negatively affect immune function (6, 7), endocrine homeostasis (8), nutrient metabolism (9, 10), and cognitive function (11, 12), and therefore contribute to a considerable burden of physical and mental health problems (13–15), including cardiovascular disease (CVD) (16, 17).

Growing epidemiological evidence supports that sleep disorders are associated with increased mortality in the general population (18–20). However, the high missed diagnosis rate of insomnia has been revealed in a recent study (4), which reported an insomnia disorder estimate of 25.2% among Australian adults using the widely accepted contemporary ICSD-3 criteria, of which only 5.5% have been ever diagnosed with insomnia. Moreover, a large number of people suffer from self-perceived sleep problems but do not meet the diagnostic criteria for sleep disorders. A multi-center survey of 22,330 adults from 13 countries (21, 22) reported that more than 35% of participants experienced insomnia symptoms, with ~17% meeting insomnia disorder criteria during the first months of the 2019 Coronavirus pandemic. Although considered an imprecise measure, subjective sleep complaints remain an important indicator of sleep health, as they may reflect the presence of sleep impairment that is difficult to identify and assess (1).

A number of studies have previously focused on the relationship between mortality and sleep problems; however, variation existed in the definition of sleep problems in the literature, with two main categories: sleep complaints (single or multiple sleep symptoms) and sleep disorders (clinical diagnosis). Compared with sleep disorders, it is unclear whether the subjective complaint of having trouble sleeping is associated with increased all-cause and heart disease mortality risk in the general population. Although progress has been made in evaluating the relationship between mortality and sleep complaints, such as insomnia symptoms, the conclusions were inconsistent (23–31). Those studies displayed considerable heterogeneity in population inclusion criteria (e.g., age, gender, or specific diseases) and duration of follow-up (ranging from years to decades). Furthermore, the joint effects of sleep duration and sleep complaints on mortality risk may also significantly affect the results. We assumed that the inconsistency in the conclusions of these articles may be partially due to the heterogeneities mentioned earlier.

Therefore, the aims of this study were to examine the relationship between sleep complaints and all-cause and heart disease mortality and whether the associations were influenced by follow-up time and disease status utilizing data from five cycles of the National Health and Nutrition Examination Survey (NHANES) (2005~2014) linked with the most updated 2019 National Death Index (NDI). In addition, we aimed to figure out the influence of the joint effects of sleep duration and sleep complaints on mortality risk.

## 2. Methods

### 2.1. Study population

All participants were selected from five cycles of “continuous NHANES” (2005~2014). Details of the NHANES have been described online (https://www.cdc.gov/nchs/index.htm). Briefly, NHANES used a complex multistage probability sampling method to collect nationally representative health-related data of the US population. Data were obtained by in-person interview and mobile physical examination. In the present study, 50,965 participants from the continuous NHANES (2005–2014) datasets were first enrolled. Then, we excluded participants who were aged < 18 years (*n* = 20,670) or with no data on mortality (*n* = 57), sleep behaviors (*n* = 110), history of CVD or cancer disease (*n* = 1,973), and other covariates (*n* = 203). Therefore, a total of 27,952 participants were included in the final analysis (Supplementary Figure S1). The NHANES has been approved by the National Center for Health Statistics Ethics Review Board. All participants gave written informed consent. The present investigation relied on deidentified publicly available data and the project was approved by the Ethics Committee of Xuanwu Hospital, Capital Medical University [approval number (2022) 127]. The study method and results were reported following the Strengthening the Reporting of Observational Studies in Epidemiology Statement for cross-sectional studies (32).

### 2.2. Data collection

Sleep complaints were determined through two questions at the baseline visit. Question 1 was “Have you ever told a doctor or other health professional that you have trouble sleeping?”, with responses “Yes”, “No”, “Refused”, or “Don't know”, and question 2 was “Have you ever been told by a doctor or other health professional that you have a sleep disorder?”, with responses “Yes”, “No”, “Refused”, or “Don't know”. Those who answered ‘Yes' to question 1 and/or question 2 were considered as having sleep complaints, those who answered ‘Yes' to question 2 were considered as having a sleep disorder, and those who answered ‘Yes' to question 1 but ‘No' to question 2 were considered as having sleep complaints only. Sleep duration was determined using the following question: “How much sleep do you get (hours)?”, and then was categorized into four groups (< 6 h, 6–8 h, 8–10 h, and ≥ 10 h/day). Baseline weight and height were measured during a mobile physical examination. For those who did not participate in the physical examination, we used the current self-reported weight and height recorded in the baseline questionnaire as an alternative. The baseline body mass index (BMI) was calculated as weight (kg) divided by the square of height (m^2^). Information on covariates was available through baseline questionnaires, including age, sex, education level (high school or below vs. college or above), smoking status (never smoker vs. former/current smoker), leisure time moderate-to-vigorous physical activity (MVPA) [meeting (≥10 MET-h/week) vs. not meeting the guideline (< 10 MET-h/week)], and history of diabetes, hypertension, CVD (including congestive heart failure, coronary heart disease, and stroke), and cancer (history of or currently suffering from any kind of cancer or malignancy). Depression was measured using the Patient Health Questionnaire-9 (PHQ-9), a nine-item self-report instrument used as a screening and diagnostic tool (33). Each instrument was given a point ranging from 0 to 3 according to the frequency of symptoms of depression over the past 2 weeks and the total PHQ-9 score ranged from 0 to 27. As recommended by a previous study (34), a PHQ-9 total score of ≥ 5 was regarded as clinically relevant depression in this study.

### 2.3. Total mortality and heart disease mortality

The outcome was the final mortality status as well as the leading cause of death until 31 December 2019 ascertained by the mortality data from the NDI. The NDI is a highly reliable resource for death identification and the method could be found from the National Center for Health Statistics (https://www.cdc.gov/nchs/data-linkage/mortality-public.htm#). The causes of death were classified according to the codes of the International Statistical Classification of Diseases, 10th Revision (ICD-10). Deaths from heart diseases were identified according to ICD-10 codes I00-I09, I11, I13, and I20-I51. Persons who survived were administratively censored on December 31, 2019. Follow-up time for each person was months between the NHANES interview date and death or the last known date alive or censored from the mortality file.

### 2.4. Statistical analyses

We accounted for complex survey design factors according to the public guidelines for using NHANES data (https://www.cdc.gov/nchs/nhanes/about_nhanes.htm), including the sample weights, stratification, and clustering. Baseline characteristics across with or without sleep complaints were presented as weighted mean (SE) for continuous variables and number (weighted percentage) for categorical variables and were compared using the Rao-Scott χ^2^ test for categorical variables and the *t*-test for continuous variables.

Cox proportional hazards models were used to estimate hazard ratios (HRs) and 95% confidence intervals (CIs) of all-cause and heart disease mortality associated with sleep complaints. We tested the proportional hazards assumption for complex survey data by creating time-dependent covariates. We first examined the relationship between sleep complaints and all-cause and heart disease mortality in all included participants. We then conducted these analyses among subgroups with or without a history of CVD or cancer, separately. We further investigated the relationship between sleep complaints and short- and long-term outcomes among the total population and the subgroup with CVD or cancer at baseline using the survival status at 2 years of follow-up or excluding individuals who died within 2 years of follow-up, respectively. A crude model and two multivariable models were constructed. Model 1 showed unadjusted results. In model 2, we adjusted for the baseline age (years, continuous), and gender. In model 3, we additionally adjusted for education level, smoking status, leisure time MVPA level, BMI, history of diabetes, and hypertension. Plots were constructed to visualize the fully adjusted (model 3) cumulative incidence of all-cause and heart disease death for participants with and without sleep complaints. Given the symptoms of depression commonly co-occur with insomnia and were associated with mortality (26), we also repeated the main analyses after adding depression (categorical variable, PHQ-9 total score ≥ 5 vs. PHQ-9 total score < 5) as a confounder in the cox models.

The combined effects of sleep duration (< 6 h, 6–8 h, 8–10 h, and ≥ 10 h/day) and sleep complaints were estimated in the multivariate Cox models. In addition, we examined the non-linear relationship between sleep duration and risk of all-cause and heart disease death with restricted cubic splines.

All statistical analyses were performed using R (version 4.1.2) and SAS (version 9.4) with survey-specific commands and a two-tailed *P*-value of < 0.05 was considered to be statistically significant.

## 3. Results

### 3.1. Baseline characteristics

A total of 27,952 participants (mean age, 46.9 years; men, 48.5%) were included in the statistical analysis. The weighted prevalence of sleep complaints was 27.2% (95% CI, 25.3–29.0%), which was much higher than that of sleep disorders (8.3% [95% CI, 7.7–8.9%]). Table 1 shows the baseline characteristics of the participants with and without sleep complaints. In general, compared with participants without sleep complaints, those who suffered from it were more likely to be older, women, former or current smokers, physically inactive, and had a shorter sleep duration, higher BMI, and higher prevalence of hypertension, diabetes mellitus, CVD, cancer, and depression.

**Table 1:** Baseline characteristics of all included participants by sleep complaints from NHANES 2005 through 2014^a^.

**Characteristics**	**Sleep complaint**		**P-value**
	**No (*****n** =* **20,845)**	**Yes (*****n** =* **7,107)**	
Age, mean (SE), y	45.6 (0.3)	50.5 (0.27)	< 0.001
**Sex**			
Male	10,594 (50.6)	2,968 (41.5)	< 0.001
Female	10,251 (49.4)	4,139 (58.5)	
**Education level**			
High school or below	10,472 (41.0)	3,367 (39.5)	0.079
College or above	10,373 (59.0)	3,740 (60.5)	
**Smoking status**			
Never smoker	11,996 (57.6)	3,277 (46.1)	< 0.001
Former/current smoker	8,849 (42.4)	3,830 (53.9)	
Hypertension	6,309 (26.4)	3,507 (43.8)	< 0.001
Diabetes mellitus	2,071 (6.9)	1,248 (13.4)	< 0.001
CVD	1,436 (5.3)	1,043 (11.1)	< 0.001
Cancer	1,640 (8.0)	951 (13.9)	< 0.001
BMI, mean (SE), kg/m^2^	28.2 (0.09)	30.0 (0.11)	< 0.001
**Leisure time MVPA level**			
low (< 10 MET-h/week)	10,415 (43.1)	3,860 (48.7)	< 0.001
high (≥10 MET-h/week)	10,430 (56.9)	3,247(51.3)	
Sleep duration, mean (SE), hours/day	7.0 (0.01)	6.5 (0.03)	< 0.001
**Sleep duration level**			
6–8h/day	10,598 (53.6)	3,375 (50.3)	< 0.001
< 6h/day	2,378 (9.7)	1,941 (23.0)	
8–10h/day	7,246 (34.3)	1,606 (24.5)	
≥10h/day	623 (2.4)	185 (2.2)	
Depression^b^	3,089 (15.4)	2,723 (40.2)	< 0.001
All-cause death	2,700 (9.4)	1,248 (13.2)	< 0.001
Heart disease-specific death	675 (2.2)	309 (3.0)	< 0.001

NHANES, National Health and Nutrition Examination Survey; CVD (cardiovascular disease, including congestive heart failure, coronary heart disease, and stroke); BMI, body mass index; MVPA, moderate to vigorous physical activity; MET, metabolic equivalent task.

a All estimates accounted for complex survey designs. Data are presented as No. (weighted %) for categorical variables and mean (SE) for continuous variables.

b 3,853 participants were excluded because of missing information on the PHQ-9 score.

### 3.2. Sleep complaints and mortality

**Figure 1:**
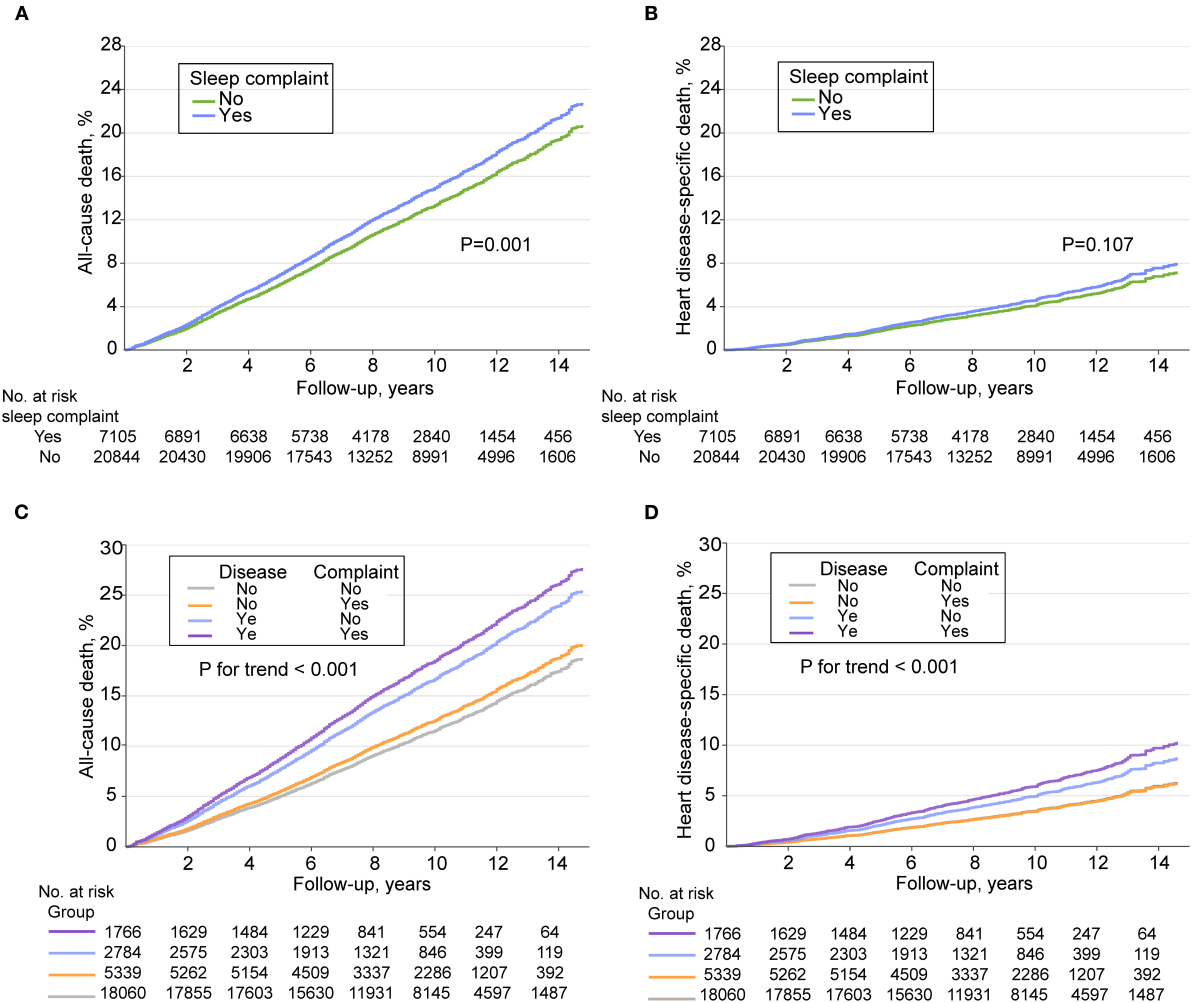
Adjusted cumulative incidence curves of all-cause and heart disease mortality among adults in the National Health and Nutrition Examination Survey 2005 through 2014. **(A)** Adjusted cumulative incidence curves of all-cause mortality stratified by sleep complaints. **(B)** Adjusted cumulative incidence curves of heart disease mortality stratified by sleep complaints. **(C)** Adjusted cumulative incidence curves of all-cause mortality stratified by cross groups of sleep complaints and disease (cardiovascular disease or cancer). **(D)** Adjusted cumulative incidence curves of heart disease mortality stratified by cross groups of sleep complaints and disease (cardiovascular disease or cancer).

During a median follow-up of 9.25 years (interquartile range, 6.75–11.75 years), 3,948 deaths occurred, including 984 deaths from heart disease. Sleep complaints were associated with all-cause and heart disease mortality in crude models (Table 2). After adjustment for age, sex, education level, smoking status, leisure time MVPA level, BMI, and history of diabetes and hypertension, the multivariable-adjusted HR of sleep complaints on all-cause mortality risk decreased but remained statistically significant (HR, 1.17; 95% CI, 1.07–1.28) (Table 2). However, the multivariable-adjusted HR of sleep complaints on heart disease mortality was no longer statistically significant (Table 2). Figures 1A, B shows the adjusted cumulative incidence curves of all-cause and heart disease mortality for sleep complaints among all included participants.

**Table 2:** Association of sleep complaints with all-cause and heart disease mortality among all participants^a^.

**HR (95% CI) and** ***P*****-value**				
**Model**	**All-cause mortality**		**Heart disease mortality**	
Model 1^b^	1.49 (1.36–1.63)	< 0.001	1.46 (1.26–1.69)	< 0.001
Model 2^c^	1.25 (1.15–1.37)	< 0.001	1.25 (1.07–1.46)	0.005
Model 3^d^	1.17 (1.07–1.28)	0.001	1.14 (0.97–1.33)	0.107

HR, hazard ratio; CI, confidence interval; MVPA, moderate–to–vigorous physical activity; BMI, body mass index.

a All estimates accounted for complex survey designs.

b Model 1 was a crude model.

c Model 2 was adjusted for age and sex.

d Model 3 was adjusted for the variables in model 2 plus education level, smoking status, leisure time MVPA level, BMI, history of diabetes, and hypertension.

### 3.3. Stratified analyses in subgroups with and without CVD or cancer

Figures 1C, D shows the adjusted cumulative incidence curves of all-cause and heart disease mortality for cross groups of sleep complaints and CVD or cancer at baseline [no sleep complaint and no CVD or cancer (group 1); isolated sleep complaints (group 2); isolated CVD or cancer (group 3); with sleep complaints and CVD or cancer (group 4)] among all included participants, using group 1 as the reference. An increasing trend of risk of all-cause and heart disease mortality from group 1 to group 4 was observed (*P* < 0.001 for trend; detailed statistical results are shown in Supplementary Table S1). When participants were stratified according to the presence or absence of CVD or cancer at baseline, sleep complaints were found to be associated with all-cause (multivariable-adjusted HR,1.17; 95% CI, 1.05–1.32) and heart disease (multivariable-adjusted HR, 1.24; 95% CI, 1.01–1.53) mortality among the group with CVD or cancer only (Table 3).

**Table 3:** Associations of sleep complaints with all-cause and heart disease mortality in subgroups with or without CVD or cancer^a^.

**Mortality**	**HR (95% CI) and** ***P*****-value**					
	**Model 1** ^b^		**Model 2** ^c^		**Model 3** ^d^	
**Subgroup without CVD or cancer at baseline**						
All-cause	1.41 (1.24–1.59)	< 0.001	1.17 (1.03–1.33)	0.013	1.10 (0.96–1.25)	0.154
Heart disease	1.28 (0.99–1.66)	0.064	1.06 (0.82–1.37)	0.664	0.99 (0.76–1.29)	0.933
**Subgroup with CVD or cancer at baseline**						
All-cause	0.95 (0.84–1.07)	0.404	1.23 (1.11–1.37)	< 0.001	1.17 (1.05–1.32)	0.006
Heart disease	0.97 (0.77–1.21)	0.792	1.34 (1.09–1.64)	0.005	1.24 (1.01–1.53)	0.037

CVD, cardiovascular disease; HR, hazard ratio; CI, confidence interval; MVPA, moderate–to–vigorous physical activity; BMI, body mass index.

a All estimates accounted for complex survey designs.

b Model 1 was a crude model.

c Model 2 was adjusted for age and sex.

d Model 3 was adjusted for the variables in model 2 plus education level, smoking status, leisure time MVPA level, BMI, history of diabetes, and hypertension.

### 3.4. Sleep complaints with short- or long-term mortality

Among all participants, sleep complaints were associated with a 1.41-fold (95% CI, 1.13–1.76) and 1.13-fold (95% CI, 1.02–1.25) increased risk of short- and long-term all-cause death, respectively, and among those with CVD or cancer at baseline, the hazard ratios were 1.37 (95% CI, 1.08–1.75) and 1.14 (95% CI, 1.00–1.29). The association between sleep complaints and short-term heart disease mortality was only evident for participants with CVD or cancer at baseline, with a multivariable-adjusted HR of 1.96 (95% CI, 1.20–3.21) (Table 4).

**Table 4:** Associations of sleep complaints with short- or long-term all-cause and heart disease mortality among all participants and subgroups with CVD or cancer^a^.

		**HR (95% CI) and** ***P*****-value**					
**Term**	**Mortality**	**Model 1** ^b^		**Model 2** ^c^		**Model 3** ^d^	
**All participants**							
Short	All-cause	1.71 (1.38–2.13)	< 0.001	1.47 (1.18–1.83)	0.001	1.41 (1.13–1.76)	0.002
	Heart disease	1.80 (1.25–2.59)	0.002	1.55 (1.06–2.25)	0.023	1.42 (0.96–2.10)	0.076
Long	All-cause	1.45 (1.32–1.60)	< 0.001	1.22 (1.11–1.34)	< 0.001	1.13 (1.02–1.25)	0.018
	Heart disease	1.41 (1.18–1.68)	< 0.001	1.20 (1.00–1.44)	0.047	1.10 (0.91–1.32)	0.338
**Participants with CVD or cancer at baseline**							
Short	All-cause	1.12 (0.87–1.44)	0.396	1.36 (1.07–1.71)	0.011	1.37 (1.08–1.75)	0.011
	Heart disease	1.61 (1.00–2.60)	0.05	2.04 (1.26–3.31)	0.004	1.96 (1.20–3.21)	0.007
Long	All-cause	0.91 (0.80–1.04)	0.178	1.21 (1.07–1.36)	0.002	1.14 (1.00–1.29)	0.048
	Heart disease	0.87 (0.66–1.14)	0.316	1.23 (0.95–1.58)	0.114	1.13 (0.88–1.45)	0.354

CVD, cardiovascular disease; HR, hazard ratio; CI, confidence interval; MVPA, moderate to vigorous physical activity; BMI, body mass index.

a All estimates accounted for complex survey designs.

b Model 1 was a crude model.

c Model 2 was adjusted for age and sex.

d Model 3 was adjusted for the variables in model 2 plus education level, smoking status, leisure time MVPA level, BMI, history of diabetes, and hypertension.

### 3.5. Sleep duration, sleep complaints, and mortality

**Figure 2:**
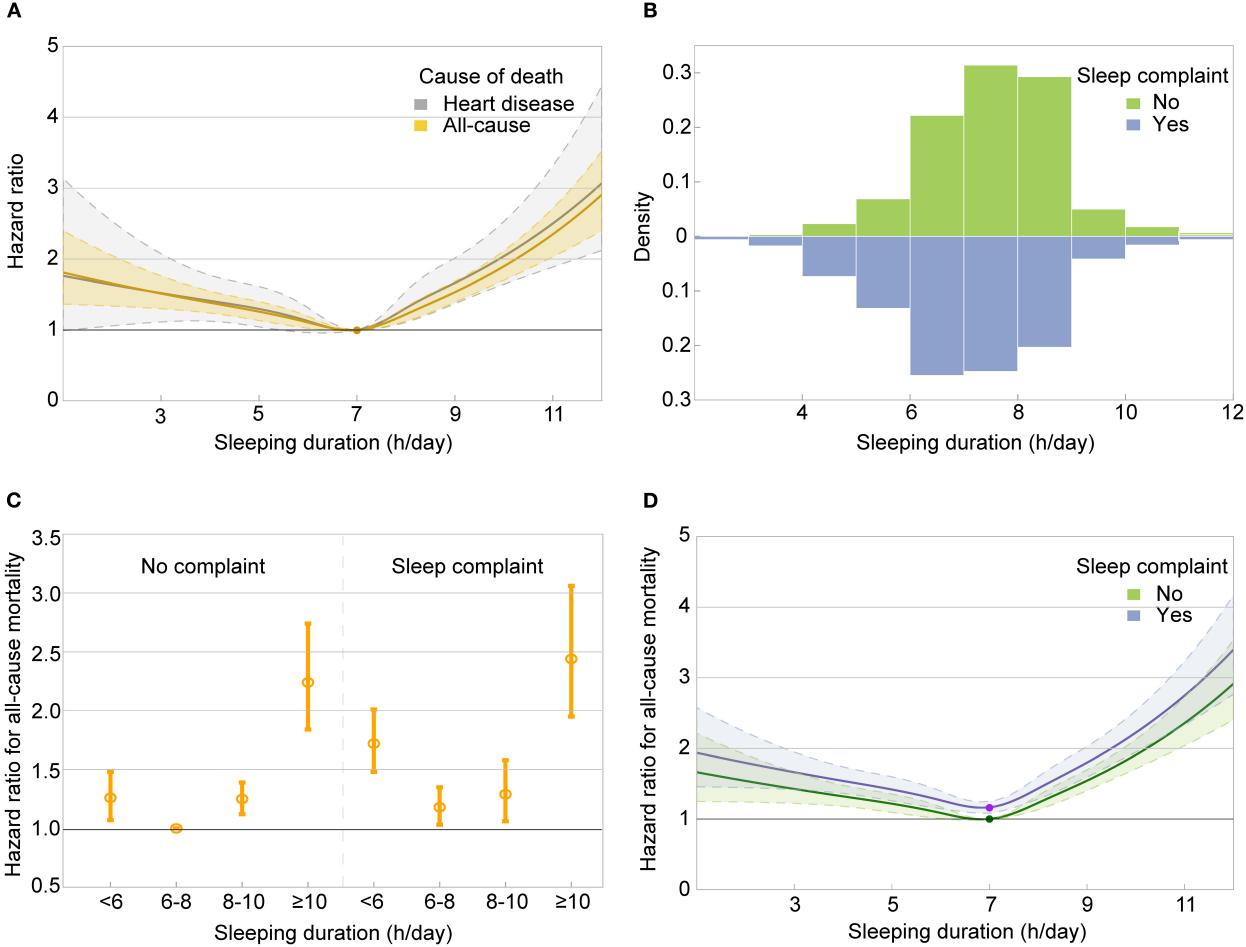
Relationship between sleep duration, sleep complaints, and mortality. **(A)** Dose–response association between sleep duration and risk of all-cause and heart disease mortality. **(B)** The distribution of sleep duration by sleep complaints. **(C)** The hazard ratios of all-cause mortality among eight combined groups of sleep duration and sleep complaints in the joint analysis. **(D)** Dose–response association between sleep duration and risk of all-cause mortality by sleep complaints. Associations in panels **(A, D)** were examined by multivariable Cox regression models based on restricted cubic splines. The solid line represents estimates of hazard ratios and the dashed line represents 95% CIs. *P*-values for non-linear association were < 0.001 in both panels. Risk estimates in panels **(A, C, D)** were adjusted for baseline age, sex, education level, smoking status, leisure time moderate-to-vigorous physical activity level, body mass index, history of diabetes, and hypertension.

The restricted cubic splines indicated a U-shaped relationship between sleep duration and all-cause or heart disease mortality among all participants, and the lowest risk was observed at the sleep duration of about 7 h/day (Figure 2A). Participants with sleep complaints had shorter sleep than those without (Figure 2B and Supplementary Table S2). In the joint analysis of sleep duration (< 6 h, 6–8 h, 8–10 h, and ≥10 h/day), as well as sleep complaints and the risk of mortality, participants sleeping ≥10 h per day with sleep complaints had the highest risk for all-cause mortality (HR, 2.44; 95% CI, 1.95–3.06), and those sleeping 6–8h per day free from sleep complaints (the reference group, HR = 1.00) had the lowest risk (Figure 2C). Of the eight combined subgroups, five subgroups had fewer than 100 deaths from heart disease during the follow-up; the joint analysis results for heart disease mortality need to be interpreted with caution due to the small statistical power (Supplementary Tables S3–S5). When participants were stratified by sleep duration, sleep complaints were associated with a higher risk of all-cause mortality only among the short (< 6 h/day; HR, 1.40; 95% CI, 1.15–1.69) or recommended (6–8 h/day; HR, 1.15; 95% CI, 1.01–1.31) sleep duration group. When participants were stratified by sleep complaints, the U-shaped association between sleep duration and all-cause mortality was consistent (Figure 2D and Supplementary Tables S3–S5). The effects of sleep complaints and sleep duration on all-cause mortality can be cumulated; however, their interaction was not statistically significant (*p* = 0.190). We repeated the main analyses of the associations between sleep complaints and all-cause and heart disease mortality after appending sleep duration (categorical variable) as a confounder in the Cox models, and there were no substantial alterations in the results (Supplementary Table S6).

### 3.6. Sleep complaints, isolated sleep complaints, sleep disorders, and mortality

We further compared the relationship of mortality with sleep complaints, isolated sleep complaints, and sleep disorders, as these three groups may reflect the severity of sleep problems to some extent. The relationship of mortality with isolated sleep complaints or sleep disorders was similar to that with sleep complaints, with the highest effect size associated with sleep disorders, the intermediate with sleep complaints, and the lowest with isolated sleep complaints in the multivariable-adjusted models (Supplementary Table S7). There were no substantial alterations when further adding sleep duration as a confounder (Supplementary Table S6).

### 3.7. Sensitivity analyses

We also conducted the main analyses after adding depression (categorical variable, PHQ-9 total score ≥ 5 vs. PHQ-9 total score < 5) as a confounder in the Cox models given the potentially complex relationships among sleep complaints, depression, and mortality (18, 20, 26). When further adjusting for depression, sleep complaints remained associated with short-term all-cause and heart disease mortality in those with CVD or cancer at baseline (Supplementary Table S8). We also explored the association of mortality with isolated sleep complaints and sleep disorders after adding depression as a confounder in the Cox models; the results were consistent with that of the main analysis, with the highest effect size associated with sleep disorders, the intermediate with sleep complaints, and the lowest with isolated sleep complaints (Supplementary Table S8).

## 4. Discussion

First, we found a significant difference in the HR of all-cause mortality for individuals with sleep complaints when compared with those without. In addition, in the stratified analyses, a 17% higher all-cause mortality risk and a 24% higher heart disease mortality risk of sleep complaints were observed among those with a history of CVD or cancer at baseline, while, by contrast, no association was statistically significant among those without. In addition, the strength of the association between sleep complaints and short-term mortality (within 2 years from baseline) was considerably stronger than that of the association between sleep complaints and long-term mortality. Therefore, our findings supported that the controversial findings in previous studies can be partially explained by significant diverseness across studies on demographic features of the study populations (e.g., the proportion of patients with CVD or cancer), and methodological issues (e.g., follow-up duration).

The causes and mechanisms underlying the discrepant associations between sleep complaints and short- or long-term mortality were not entirely clear. The relationship between sleep complaints and elevated short-term mortality risk might be partly attributable to the participant's medical conditions. That is, sleep problems might simply be a concomitant symptom of a health condition rather than act as a risk factor. Therefore, the association between sleep problems and long-term mortality risk might be more convincing for inferring a causal association between sleep problems and mortality risk. The results of this study supported an association between sleep complaints and long-term all-cause mortality risk, but the association might be underestimated due to the misclassification bias caused by the decreasing representativeness of baseline characteristics for the entire follow-up period as the follow-up period increases. The association between sleep complaints and long-term mortality risk needs to be further investigated in longitudinal studies using more finely grained repeated recordings of sleep profiles and other covariates. Although the short-term association between sleep complaints and mortality risk may be less robust for causal inference, the association found in this study between sleep complaints and short-term all-cause and heart disease mortality risk in people with CVD or cancer at baseline suggested, at a minimum, that the presence of sleep complaints in people with CVD or cancer may act as a “label”, indicating that individuals with sleep complaints in this particular group are at greater risk of short-term death and require additional attention and management.

In contrast to the inconsistent results of sleep complaints, the U-shaped association between sleep duration and mortality has been recognized by strong evidence from prospective studies (35–41) and the consensus statement from the American Academy of Sleep Medicine and Sleep Research Society (42). However, few studies have focused on the pattern of joint effects of sleep duration and sleep complaints on mortality risk, having fully considered the U-shaped association between sleep duration and mortality risk. Hedström et al. (28) reported that the U-shaped association between sleep duration and increased mortality risk appeared restricted to those with insomnia symptoms (28), which was partially inconsistent with the study by Chien et al. (43) as they did not observe a significant U-shaped association between sleep duration and mortality in either the infrequent insomnia group or the frequent insomnia group (43). In this study, the U-shaped association between sleep duration and all-cause mortality risk was observed both in participants with and without sleep complaints. In addition, the two studies mentioned above concluded that the association between insomnia and mortality is only evident among long sleepers (≥9 h/day), while we found that sleep complaints were associated with a higher risk of all-cause mortality only among the short (< 6 h/day) or recommended (6–8 h/day) sleep duration groups in the present study. Although the sample size of the Swedish National March Cohort (SNMC) (28) and this study were both larger than 20,000 people, obvious variation in baseline characteristics also existed between the two populations. In particular, the SNMC had a higher prevalence of sleep complaints than that of this study (75.3 vs. 27.2%), and the proportions of the short (< 6 h/day) and recommended (6–8 h/day) sleep duration groups in the SNMC cohort were 8.2% and 89.5%, while in this cohort, they were 15.5% and 50.0%, respectively. An international multi-center harmonized survey published the findings that of the 22,330 general adults from 13 countries and four continents, 36.7% had clinical insomnia symptoms (22). The sleep characteristics of this study were closer to the aforementioned multi-center study. Of note, in this study, the short sleep duration group (< 6 h/day, 47.0%) had the highest prevalence of sleep complaints, followed by the recommended group (6–8 h/day, 25.9%), which is consistent with the study by Hedström et al. (28). These findings increase the need to focus on the subgroup with sleep complaints even though they may have the optimal sleep duration.

The relationships between depression, sleep problems, and mortality risk are complex (18, 20, 26). In the study by Leggett et al., (26) the authors concluded that both insomnia symptoms and depressive symptoms are associated with a greater risk of death; however, depressive symptoms accounted for the insomnia association when both were considered in the model (26). The purpose of the present study was to determine the relationship between mortality and sleep complaints, and we observed that the independent associations were statistically significant, even when further adjusted for depression (PHQ-9 ≥5). In the current study, the weighted mean (SE) of the PHQ-9 score in subjects with sleep disorders or complaints was 5.5 (0.17) and 5.0 (0.10), respectively. The PHQ-9 score of the sleep complaints group was lower than that of the sleep disorder group. The findings of this study suggest that the relationship between sleep complaints and a higher risk of short-term all-cause and heart disease mortality in a population with mild depressive symptoms cannot be fully explained by depression, which was supported by the finding from a recent study concluding that clinical insomnia was independently associated with an increased risk of recurrent major adverse cardiovascular events in patients with coronary heart disease, even after adjustment for depression (44).

The present study had several limitations. First, owing to the cross-sectional nature of the NHANES study, the survival analyses were based on baseline sleep behaviors assessment, which may not accurately reflect the long-term status. Second, covariates collected at baseline may also change over time. There might be a complex interplay between covariates that can change over time and sleep complaints. Third, residual or unknown confounders cannot be entirely excluded, although a multitude of potential confounding factors both associated with sleep and mortality have been taken into consideration. Fourth, the identification of sleep complaints and disorders was dependent on the answers to questions in a sleep questionnaire completed in the home using the Computer-assisted Personal Interview (CAPI) system. The CAPI was programmed with built-in consistency checks to reduce data entry errors. The CAPI also uses online help screens to assist interviewers define key terms used in the questionnaire. Therefore, the answers were reliable but still lacked validation from objective examinations and information about the specific diagnosis (e.g., obstructive sleep apnea), quantitative statistics of sleep disorders, as well as whether sleep problems are primary or secondary. Finally, we did not adjust the use of sleep medication in the multivariate Cox models. However, assuming that having sleep complaints is a risk factor for death, the protective effects of sleep drugs will bias the effect to negative results. Even so, we still found that sleep complaints were associated with a higher risk of all-cause mortality and heart disease mortality, indicating that the positive results in this study were reliable, but the effect size might be underestimated.

Overall, sleep complaints at baseline were positively associated with a higher risk of all-cause mortality and heart disease mortality, particularly for those with a history of CVD or cancer at baseline, and the association persisted after further adjustment for sleep duration and depression. Even among people who sleep within the recommended duration, sleep complaints remained associated with all-cause mortality. These findings emphasize the importance of managing sleep complaints, particularly in those with CVD and cancer.

## Data availability statement

The original datasets used in this study are publicly available, and they can be found at: https://www.cdc.gov/nchs/index.htm.

## Ethics statement

The studies involving human participants were reviewed and approved by the Ethics Committee of Xuanwu Hospital, Capital Medical University. The NHANES has been approved by the National Center for Health Statistics Ethics Review Board. All participants gave written informed consent.

## Author contributions

YW and LR contributed to the study design and manuscript edits. QW and SH contributed to the data analysis and data interpretation and drafted the manuscript. NP and TZ contributed to the data interpretation and manuscript edits. All authors contributed to the article and approved the submitted version.
