# Supplementary material for: Association of sleep complaints with all-cause and heart disease mortality among US adults
Source: Front Public Health. 2023 Mar 21;11:1043347. doi: 10.3389/fpubh.2023.1043347 (PMC10070800; doi:10.3389/fpubh.2023.1043347)
Supplement: Supplementary file 1 [file Table_1.DOCX]

Supplementary Material

**Supplementary Table 1**

Associations of sleep complaint with all-cause and heart disease mortality for participants by cross groups of sleep complaint and CVD or cancer among all included participants^a^.

|  | Category | | Model 1^b^ | | | Model 2^c^ | | | Model 3^d^ | | |
| --- | --- | --- | --- | --- | --- | --- | --- | --- | --- | --- | --- |
| Mortality | Sleep complaint | CVD or cancer | HR (95% CI) | *P* | P for trend | HR (95% CI) | *P* | P for trend | HR (95% CI) | *P* | P for trend |
| All-cause | No | No | Reference | / | <0.001 | Reference | / | <0.001 | Reference | / | <0.001 |
|  | Yes | No | 1.40(1.24-1.59) | <0.001 |  | 1.16(1.02-1.32) | 0.019 |  | 1.11(0.97-1.27) | 0.115 |  |
|  | No | Yes | 6.21(5.65-6.81) | <0.001 |  | 1.69(1.55-1.84) | <0.001 |  | 1.63(1.49-1.78) | <0.001 |  |
|  | Yes | Yes | 5.89(5.20-6.67) | <0.001 |  | 2.11(1.86-2.39) | <0.001 |  | 1.88(1.67-2.13) | <0.001 |  |
| Heart disease | No | No | Reference | / | <0.001 | Reference | / | <0.001 | Reference | / | <0.001 |
|  | Yes | No | 1.27(0.98-1.65) | 0.067 |  | 1.05(0.81-1.37) | 0.697 |  | 0.99(0.76-1.29) | 0.941 |  |
|  | No | Yes | 7.04(5.92-8.38) | <0.001 |  | 1.55(1.31-1.83) | <0.001 |  | 1.48(1.26-1.74) | <0.001 |  |
|  | Yes | Yes | 6.84(5.70-8.21) | <0.001 |  | 2.10(1.73-2.55) | <0.001 |  | 1.82(1.50-2.21) | <0.001 |  |

Abbreviations: CVD, cardiovascular disease; HR, hazard ratio; CI, confidence interval; MVPA, moderate-to-vigorous physical activity; BMI, body mass index.

^a^ All estimates accounted for complex survey designs.

^b^ Model 1 was a crude model.

^c^ Model 2 was adjusted for age, sex.

^d^ Model 3 was adjusted for the variables in model 2 plus education level, smoking status, leisure time MVPA level, BMI, history of diabetes and hypertension.
