# Supplementary material for: Association of sleep complaints with all-cause and heart disease mortality among US adults
Source: Front Public Health. 2023 Mar 21;11:1043347. doi: 10.3389/fpubh.2023.1043347 (PMC10070800; doi:10.3389/fpubh.2023.1043347)
Supplement: Supplementary file 2 [file Table_2.DOCX]

Supplementary Material

**Supplementary Table 2**

The distribution of sleep complaint among four sleep duration groups (<6h, 6–8h, 8–10h, and ≥10h/day)^a^.

|  | Sleep duration | | | |  |
| --- | --- | --- | --- | --- | --- |
| Sleep complaint | <6 h/day | 6-8h/day | 8-10 h/day | ≥10 h/day | *P* |
| No | 2378(11.4) | 10598(50.8) | 7246(34.8) | 623(3.0) | <0.001 |
| Yes | 1941(27.3) | 3375(47.5) | 1606(22.6) | 185(2.6) |  |

^a^ All estimates accounted for complex survey designs. Data were presented as number (weighted percentage).
