# Supplementary material for: Association of sleep complaints with all-cause and heart disease mortality among US adults
Source: Front Public Health. 2023 Mar 21;11:1043347. doi: 10.3389/fpubh.2023.1043347 (PMC10070800; doi:10.3389/fpubh.2023.1043347)
Supplement: Supplementary file 3 [file Table_3.DOCX]

Supplementary Material

**Supplementary Table 3**

The joint effects of sleep duration and sleep complaint for all-cause and heart disease mortality among all included participants^a^.

| mortality | Combined groups | | No. of subjects | No. of events | HR (95% CI)^b^ | p |
| --- | --- | --- | --- | --- | --- | --- |
|  | Sleep duration | Sleep complaint |  |  |  |  |
| All-cause | 6-8 h | No | 10598 | 1042 | 1.00 (Reference) | / |
|  | <6 h | No | 2378 | 275 | 1.26(1.07-1.48) | 0.005 |
|  | 8-10 h | No | 7246 | 1177 | 1.25(1.12-1.39) | <0.001 |
|  | ≥10h | No | 623 | 206 | 2.24(1.84-2.74) | <0.001 |
|  | <6 h | Yes | 1941 | 382 | 1.72(1.48-2.01) | <0.001 |
|  | 6-8 h | Yes | 3375 | 504 | 1.18(1.03-1.35) | 0.015 |
|  | 8-10 h | Yes | 1606 | 296 | 1.29(1.06-1.58) | 0.013 |
|  | ≥10h | Yes | 185 | 66 | 2.44(1.95-3.06) | <0.001 |
| Heart disease | 6-8 h | No | 10598 | 249 | 1.00 (Reference) | / |
|  | <6 h | No | 2378 | 55 | 1.12(0.76-1.66) | 0.568 |
|  | 8-10 h | No | 7246 | 318 | 1.33(1.11-1.61) | 0.003 |
|  | ≥10h | No | 623 | 53 | 2.2(1.46-3.31) | <0.001 |
|  | <6 h | Yes | 1941 | 95 | 1.69(1.24-2.3) | 0.001 |
|  | 6-8 h | Yes | 3375 | 124 | 1.24(0.92-1.66) | 0.152 |
|  | 8-10 h | Yes | 1606 | 75 | 1.24(0.9-1.72) | 0.185 |
|  | ≥10h | Yes | 185 | 15 | 2.03(1.15-3.6) | 0.015 |

Abbreviations: HR, hazard ratio; CI, confidence interval; MVPA, moderate-to-vigorous physical activity; BMI, body mass index.

^a^ All estimates accounted for complex survey designs.

^b^ Adjusted for age, sex, education level, smoking status, leisure time MVPA level, BMI, history of diabetes and hypertension.
