# Supplementary material for: Association of sleep complaints with all-cause and heart disease mortality among US adults
Source: Front Public Health. 2023 Mar 21;11:1043347. doi: 10.3389/fpubh.2023.1043347 (PMC10070800; doi:10.3389/fpubh.2023.1043347)
Supplement: Supplementary file 4 [file Table_4.DOCX]

Supplementary Material

**Supplementary Table 4**

Associations of sleep complaint with all-cause and heart disease mortality among subgroups stratified by sleep duration^a^.

| mortality | Combined groups | | No. of subjects | No. of events | HR (95% CI)^b^ | p |
| --- | --- | --- | --- | --- | --- | --- |
|  | Sleep duration | Sleep complaint |  |  |  |  |
| All-cause | <6 h | No | 2378 | 275 | 1.00 (Reference) | / |
|  |  | Yes | 1941 | 382 | 1.40(1.15-1.69) | 0.001 |
|  | 6-8 h | No | 10598 | 1042 | 1.00 (Reference) | / |
|  |  | Yes | 3375 | 504 | 1.15(1.01-1.31) | 0.036 |
|  | 8-10 h | No | 7246 | 1177 | 1.00 (Reference) | / |
|  |  | Yes | 1606 | 296 | 1.06(0.88-1.26) | 0.551 |
|  | ≥10h | No | 623 | 206 | 1.00 (Reference) | / |
|  |  | Yes | 185 | 66 | 1.23(0.94-1.61) | 0.135 |
| Heart disease | <6 h | No | 2378 | 55 | 1.00 (Reference) | / |
|  |  | Yes | 1941 | 95 | 1.63(1.1-2.41) | 0.015 |
|  | 6-8 h | No | 10598 | 249 | 1.00 (Reference) | / |
|  |  | Yes | 3375 | 124 | 1.20(0.89-1.61) | 0.233 |
|  | 8-10 h | No | 7246 | 318 | 1.00 (Reference) | / |
|  |  | Yes | 1606 | 75 | 0.93(0.66-1.32) | 0.684 |
|  | ≥10h | No | 623 | 53 | 1.00 (Reference) | / |
|  |  | Yes | 185 | 15 | 1.02(0.47-2.18) | 0.965 |

Abbreviations: HR, hazard ratio; CI, confidence interval; MVPA, moderate-to-vigorous physical activity; BMI, body mass index.

^a^ All estimates accounted for complex survey designs.

^b^ Adjusted for age, sex, education level, smoking status, leisure time MVPA level, BMI, history of diabetes and hypertension.
