# Supplementary material for: Association of sleep complaints with all-cause and heart disease mortality among US adults
Source: Front Public Health. 2023 Mar 21;11:1043347. doi: 10.3389/fpubh.2023.1043347 (PMC10070800; doi:10.3389/fpubh.2023.1043347)
Supplement: Supplementary file 5 [file Table_5.DOCX]

Supplementary Material

**Supplementary Table 5**

Associations of sleep duration with all-cause and heart disease mortality among subgroups stratified by sleep complaint^a^.

| mortality | Combined groups | | No. of subjects | No. of events | HR (95% CI)^b^ | p |
| --- | --- | --- | --- | --- | --- | --- |
|  | Sleep duration | Sleep complaint |  |  |  |  |
| All-cause | 6-8 h | No | 10598 | 1042 | 1.00 (Reference) | / |
|  | <6 h | No | 2378 | 275 | 1.27(1.08-1.5) | 0.003 |
|  | 8-10 h | No | 7246 | 1177 | 1.22(1.1-1.36) | <0.001 |
|  | ≥10h | No | 623 | 206 | 2.18(1.76-2.7) | <0.001 |
|  | 6-8 h | Yes | 3375 | 504 | 1.00 (Reference) | / |
|  | <6 h | Yes | 1941 | 382 | 1.42(1.24-1.63) | <0.001 |
|  | 8-10 h | Yes | 1606 | 296 | 1.1(0.9-1.35) | 0.35 |
|  | ≥10h | Yes | 185 | 66 | 2.12(1.69-2.68) | <0.001 |
| Heart disease | 6-8 h | No | 10598 | 249 | 1.00 (Reference) | / |
|  | <6 h | No | 2378 | 55 | 1.13(0.76-1.68) | 0.544 |
|  | 8-10 h | No | 7246 | 318 | 1.31(1.09-1.58) | 0.004 |
|  | ≥10h | No | 623 | 53 | 2.15(1.4-3.3) | <0.001 |
|  | 6-8 h | Yes | 3375 | 124 | 1.00 (Reference) | / |
|  | <6 h | Yes | 1941 | 95 | 1.31(0.91-1.88) | 0.143 |
|  | 8-10 h | Yes | 1606 | 75 | 1.01(0.69-1.5) | 0.942 |
|  | ≥10h | Yes | 185 | 15 | 1.65(0.86-3.15) | 0.133 |

Abbreviations: HR, hazard ratio; CI, confidence interval; MVPA, moderate-to-vigorous physical activity; BMI, body mass index.

^a^ All estimates accounted for complex survey designs.

^b^ Adjusted for age, sex, education level, smoking status, leisure time MVPA level, BMI, history of diabetes and hypertension.
