# Supplementary material for: Association of sleep complaints with all-cause and heart disease mortality among US adults
Source: Front Public Health. 2023 Mar 21;11:1043347. doi: 10.3389/fpubh.2023.1043347 (PMC10070800; doi:10.3389/fpubh.2023.1043347)
Supplement: Supplementary file 6 [file Table_6.DOCX]

Supplementary Material

**Supplementary Table 6**

Associations of sleep complaint, isolate sleep complaint, and sleep disorder with all-cause and heart disease mortality after adding sleep duration as a confounder^a^.

|  | Sleep complaint | | Isolate sleep complaint | | Sleep disorder | |
| --- | --- | --- | --- | --- | --- | --- |
|  | HR (95% CI)^b^ | *P* value | HR (95% CI)^b^ | *P* value | HR (95% CI)^b^ | *P* value |
| All participants | | | | | | |
| All-cause | 1.16(1.05-1.27) | 0.003 | 1.11(0.99-1.25) | 0.068 | 1.28(1.12-1.47) | <0.001 |
| Heart disease | 1.15(0.97-1.35) | 0.104 | 1.11(0.90-1.37) | 0.327 | 1.24(1.00-1.54) | 0.051 |
| Participants with CVD or cancer at baseline | | | | | | |
| All cause | 1.16(1.03-1.31) | 0.018 | 1.15(0.99-1.34) | 0.060 | 1.19(0.99-1.44) | 0.059 |
| Heart disease | 1.19(0.96-1.48) | 0.117 | 1.16(0.87-1.55) | 0.309 | 1.27(0.95-1.70) | 0.113 |
| All participants | | | | | | |
| Short-term all cause | 1.43(1.13-1.82) | 0.003 | 1.41(1.03-1.93) | 0.033 | 1.52(1.13-2.05) | 0.006 |
| Short-term heart disease | 1.44(0.96-2.15) | 0.076 | 1.42(0.91-2.20) | 0.119 | 1.28(0.71-2.29) | 0.416 |
| Long-term all cause | 1.12(1.01-1.24) | 0.038 | 1.07(0.95-1.20) | 0.255 | 1.25(1.07-1.45) | 0.004 |
| Long-term heart disease | 1.10(0.91-1.33) | 0.322 | 1.06(0.84-1.34) | 0.629 | 1.24(0.94-1.62) | 0.126 |
| Participants with CVD or cancer at baseline | | | | | | |
| Short-term all cause | 1.41(1.07-1.86) | 0.014 | 1.38(0.97-1.95) | 0.071 | 1.44(1.01-2.07) | 0.046 |
| Short-term heart disease | 1.87(1.12-3.12) | 0.017 | 1.77(1.02-3.07) | 0.041 | 1.73(0.84-3.57) | 0.135 |
| Long-term all cause | 1.11(0.97-1.27) | 0.117 | 1.11(0.95-1.30) | 0.172 | 1.15(0.93-1.41) | 0.189 |
| Long-term heart disease | 1.08(0.83-1.40) | 0.578 | 1.05(0.75-1.48) | 0.775 | 1.19(0.83-1.70) | 0.340 |

Abbreviations: CVD, cardiovascular disease; HR, hazard ratio; CI, confidence interval; MVPA, moderate-to-vigorous physical activity; BMI, body mass index.

^a^ All estimates accounted for complex survey designs.

^b^ Adjusted for age, sex, education level, smoking status, leisure time MVPA level, BMI, history of diabetes and hypertension, and sleep duration (categorical variable).
