# Supplementary material for: Association of sleep complaints with all-cause and heart disease mortality among US adults
Source: Front Public Health. 2023 Mar 21;11:1043347. doi: 10.3389/fpubh.2023.1043347 (PMC10070800; doi:10.3389/fpubh.2023.1043347)
Supplement: Supplementary file 7 [file Table_7.DOCX]

Supplementary Material

**Supplementary Table 7**

Associations of sleep complaint, isolate sleep complaint and sleep disorder with all-cause and heart disease mortality^a^.

|  | Sleep complaint | | Isolate sleep complaint | | Sleep disorder | |
| --- | --- | --- | --- | --- | --- | --- |
|  | HR (95% CI)^b^ | *P* value | HR (95% CI)^b^ | *P* value | HR (95% CI)^b^ | *P* value |
| All participants | | | | | | |
| All-cause | 1.17(1.07-1.28) | 0.001 | 1.11(1.00-1.24) | 0.053 | 1.31(1.15-1.49) | <0.001 |
| Heart disease | 1.14(0.97-1.33) | 0.107 | 1.09(0.89-1.34) | 0.387 | 1.23(0.99-1.53) | 0.062 |
| Participants with CVD or cancer at baseline | | | | | | |
| All cause | 1.17(1.05-1.32) | 0.006 | 1.14(1.00-1.3) | 0.048 | 1.24(1.02-1.51) | 0.028 |
| Heart disease | 1.24(1.01-1.53) | 0.037 | 1.21(0.92-1.58) | 0.165 | 1.30(0.96-1.77) | 0.086 |
| All participants | | | | | | |
| Short-term all cause | 1.41(1.13-1.76) | 0.002 | 1.34(1.00-1.79) | 0.049 | 1.59(1.19-2.13) | 0.002 |
| Short-term heart disease | 1.42(0.96-2.10) | 0.076 | 1.39(0.91-2.13) | 0.126 | 1.27(0.72-2.25) | 0.413 |
| Long-term all cause | 1.13(1.02-1.25) | 0.018 | 1.08(0.96-1.2) | 0.198 | 1.26(1.09-1.46) | 0.002 |
| Long-term heart disease | 1.10(0.91-1.32) | 0.338 | 1.05(0.83-1.32) | 0.699 | 1.22(0.93-1.61) | 0.148 |
| Participants with CVD or cancer at baseline | | | | | | |
| Short-term all cause | 1.37(1.08-1.75) | 0.011 | 1.31(0.97-1.76) | 0.077 | 1.47(1.02-2.11) | 0.036 |
| Short-term heart disease | 1.96(1.20-3.21) | 0.007 | 1.92(1.12-3.26) | 0.017 | 1.77(0.90-3.46) | 0.097 |
| Long-term all cause | 1.14(1.00-1.29) | 0.048 | 1.11(0.96-1.28) | 0.158 | 1.20(0.97-1.48) | 0.097 |
| Long-term heart disease | 1.13(0.88-1.45) | 0.354 | 1.09(0.79-1.50) | 0.612 | 1.22(0.85-1.76) | 0.278 |

Abbreviations: CVD, cardiovascular disease; HR, hazard ratio; CI, confidence interval; MVPA, moderate-to-vigorous physical activity; BMI, body mass index.

^a^ All estimates accounted for complex survey designs.

^b^ Adjusted for age, sex, education level, smoking status, leisure time MVPA level, BMI, history of diabetes and hypertension.
