# Supplementary material for: Association of sleep complaints with all-cause and heart disease mortality among US adults
Source: Front Public Health. 2023 Mar 21;11:1043347. doi: 10.3389/fpubh.2023.1043347 (PMC10070800; doi:10.3389/fpubh.2023.1043347)
Supplement: Supplementary file 8 [file Table_8.DOCX]

Supplementary Material

**Supplementary Table 8**

Associations of sleep complaint, isolate sleep complaint, and sleep disorder with all-cause and heart disease mortality after adding sleep duration and depression as confounders^a^.

|  | Sleep complaint | | Isolate sleep complaint | | Sleep disorder | |
| --- | --- | --- | --- | --- | --- | --- |
|  | HR (95% CI)^b^ | *P* value | HR (95% CI)^b^ | *P* value | HR (95% CI)^b^ | *P* value |
| All participants | | | | | | |
| All-cause | 1.12(1.01-1.25) | 0.033 | 1.07(0.94-1.21) | 0.298 | 1.24(1.07-1.44) | 0.004 |
| Heart disease | 1.07(0.87-1.32) | 0.534 | 1.06(0.82-1.36) | 0.666 | 1.08(0.82-1.42) | 0.589 |
| Participants with CVD or cancer at baseline | | | | | | |
| All cause | 1.14(0.98-1.33) | 0.080 | 1.13(0.95-1.33) | 0.167 | 1.15(0.94-1.42) | 0.173 |
| Heart disease | 1.11(0.85-1.44) | 0.445 | 1.10(0.78-1.56) | 0.570 | 1.13(0.81-1.56) | 0.475 |
| All participants | | | | | | |
| Short-term all cause | 1.42(1.09-1.84) | 0.010 | 1.37(1.01-1.87) | 0.045 | 1.51(1.03-2.20) | 0.033 |
| Short-term heart disease | 1.37(0.81-2.31) | 0.237 | 1.36(0.78-2.39) | 0.278 | 1.08(0.49-2.38) | 0.849 |
| Long-term all cause | 1.08(0.97-1.22) | 0.169 | 1.03(0.91-1.17) | 0.662 | 1.20(1.02-1.42) | 0.030 |
| Long-term heart disease | 1.03(0.82-1.30) | 0.803 | 1.01(0.77-1.33) | 0.924 | 1.07(0.77-1.49) | 0.667 |
| Participants with CVD or cancer at baseline | | | | | | |
| Short-term all cause | 1.50(1.08-2.08) | 0.016 | 1.41(0.96-2.09) | 0.082 | 1.52(0.91-2.53) | 0.109 |
| Short-term heart disease | 2.04(1.08-3.87) | 0.029 | 1.90(0.98-3.67) | 0.057 | 1.76(0.64-4.83) | 0.272 |
| Long-term all cause | 1.09(0.93-1.28) | 0.289 | 1.08(0.91-1.29) | 0.374 | 1.10(0.88-1.38) | 0.401 |
| Long-term heart disease | 1.00(0.74-1.35) | 0.994 | 1.00(0.68-1.48) | 0.995 | 1.05(0.73-1.51) | 0.798 |

Abbreviations: CVD, cardiovascular disease; HR, hazard ratio; CI, confidence interval; MVPA, moderate-to-vigorous physical activity; BMI, body mass index.

^a^ All estimates accounted for complex survey designs, and 3853 participants were additionally excluded because of missing information on PHQ-9 score.

^b^ Adjusted for age, sex, education level, smoking status, leisure time MVPA level, BMI, history of diabetes and hypertension, sleep duration (categorical variable), and depression (categorical variable).
