# Supplementary material for: Association of sleep complaints with all-cause and heart disease mortality among US adults
Source: Front Public Health. 2023 Mar 21;11:1043347. doi: 10.3389/fpubh.2023.1043347 (PMC10070800; doi:10.3389/fpubh.2023.1043347)

Supplementary Material

Supplementary Figure 1. Selection of study participants from the 2005 to 2014 cycles of the National Health and Nutrition Examination Survey (NHANES).

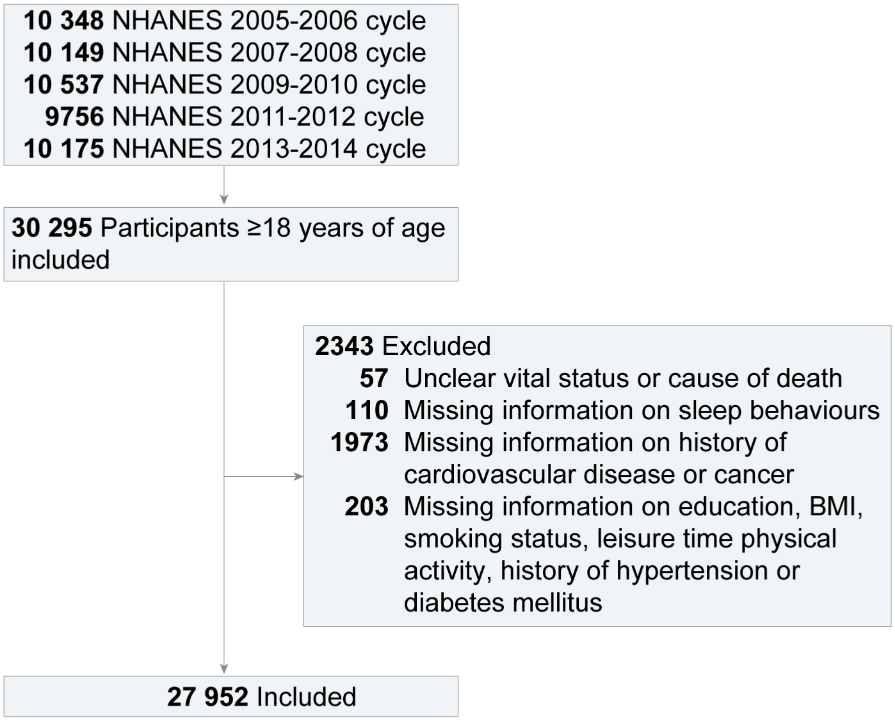

Supplement: Supplementary file 9 [file Image_1.PDF]
